# Reemergence of dengue virus type-3 (subtype-III) in India: Implications for increased incidence of DHF & DSS

**DOI:** 10.1186/1743-422X-3-55

**Published:** 2006-07-06

**Authors:** Paban Kumar Dash, Man Mohan Parida, Parag Saxena, Ajay Abhyankar, CP Singh, KN Tewari, Asha Mukul Jana, K Sekhar, PV Lakshmana Rao

**Affiliations:** 1 Division of Virology, Defence R&D Establishment, Jhansi Road, Gwalior- 474002, MP, India; 2 Municipal Corporation, Delhi-110001, India

## Abstract

**Background:**

Dengue virus infection has recently taken endemic proportion in India implicating all the four known dengue serotypes. There was a major dengue outbreak in northern India including Delhi in October- December, 2003 and again in 2004. We have carried out a detailed investigation of the 2004 outbreak by Serosurveillance, RT-PCR, nested PCR, virus isolation and genotyping. We also report the molecular epidemiological investigation of these outbreaks.

**Results:**

The serological investigation of 162 suspected serum samples using an in-house dengue dipstick ELISA revealed 11%-IgM, 51%-IgG and 38%-both IgM and IgG antibody positivity. The RT-PCR analysis revealed presence of dengue RNA in 17 samples. Further subtyping and genotyping by nested PCR and nucleotide sequencing of C-prM gene junction revealed the association of subtype III of dengue virus type 3 in the outbreak.

**Conclusion:**

The sudden shifting and dominance of the dengue virus serotype-3 (subtype III) replacing the earlier circulating serotype-2 (subtype IV) is a point of major concern and may be attributed to increased incidence of DHF and DSS in India.

## Background

Dengue virus infection is now recognized as one of the most important mosquito borne human infections of 21^st ^century. The global incidences of the dengue infection has now increased enormously and an estimated 50–100 million cases of dengue infections are now reported annually from more than 100 tropical and sub tropical countries of the world [1]. Dengue is caused by four antigenically distinct viruses designated as dengue virus type 1–4 (DEN 1–4), belonging to genus *Flavivirus *of family *Flaviviridae*. The genome of dengue virus consists of a single stranded, non segmented, positive sense ribonucleic acid (RNA) of approximately 10.7 kb in length [2]. All the four serotypes of dengue viruses are primarily transmitted by *Aedes aegypti *.Infection with any one of these serotypes generally leads to a mild, self limiting febrile illness (classical dengue fever (DF)). However, in few cases DF also leads to severe life threatening dengue hemorrhagic fever (DHF) and dengue shock syndrome (DSS). Several hypotheses, like antibody dependent enhancement (ADE) in heterotypic secondary dengue infections, involvement of a virulent viral genotype, and host factors have been suggested to explain the mechanism of pathogenesis of DHF and DSS [3].

The number of DHF and DSS cases have increased enormously in the last two decades in India and DEN-2 has been implicated as the causative agent in most of these outbreaks [4,5]. It is widely reported that DEN-2 is circulating predominantly in most parts of India and involvement of other serotypes in major dengue outbreaks are not reported since 1995. However, surprisingly, a major epidemic struck in many parts of northern India including National Capital Delhi and Gwalior in Madhya Pradesh in 2003, in which DEN-3 virus was implicated as the major serotype [6,7]. Again dengue cases were reported during September – October, 2004 in Delhi.

In the present study, we report the serological, virological and molecular investigation of the 2004 Dengue outbreak. We also report the molecular epidemiological investigation of the 2003 and 2004 Delhi outbreaks based on the nucleotide sequence analysis of C-prM gene junction.

## Results

### Outbreak

An outbreak of febrile illness was reported in Delhi, India, during September- October 2004. The trend of the epidemic indicated the maximum number of cases was reported from the 1^st ^to 3^rd ^week of October. The clinical history revealed that all the patients had suffered from fever ranging from 38.5° to 40°C. Most of the prominent clinical symptoms include headache (75%), myalgia (66%), rash (48%), vomiting (42%), conjunctival hemorrhage (38%), epistaxis (17%) and melena (5%). The platelet count varies from 18000 – 2.8 lakhs (Mean 62,000). The epidemic affected males and females at a ratio of 2.6:1. Majority (52.5%) of the patients were found belong to the age group more than 25 years. The detail distribution of the disease in terms of the age and sex of the patients is listed in Table 1.

**Table 1:** Age and sex distribution of dengue suspected patients in Delhi during September-October, 2004

Age (Year)	No. of patients		
	
	Male	Female	Total
0–5	4 (3.4%)	3(2%)	7
6–10	4(3.4%)	-	4
11–15	8(5%)	7(4.32%)	15
16–20	15 (9.25%)	5(3.08%)	20
21–25	23(14.1%)	8(5%)	31
>25	63(39%)	22(13.5%)	85

### Serology

The serological analysis revealed that a total of 141 samples (87%) are positive for the presence of dengue specific antibodies. Out of these antibody positive cases, 16 (11%) were found positive for IgM, 72 (51%) for IgG and 53 (38%) had both IgM and IgG antibodies.

### RT-PCR

A total of 17 (10%) samples were found positive for the presence of dengue virus specific nucleic acid as demonstrated by the presence of dengue complex specific 511 bp amplicon in 2% agarose gel.

### Isolation

Isolation of virus was attempted from all the RT-PCR positive samples in C6/36 cells. A total of four dengue viruses were isolated from these samples. The isolation was confirmed at each passage level by RT-PCR.

### Typing of viruses

The serotype of the isolated virus, as well as viruses directly from serum samples was determined by nested PCR using serotype specific primers. The result indicated that all the 17 samples were positive for DEN-3 specific RNA.

### Nucleotide sequence analysis

**Figure 1:**
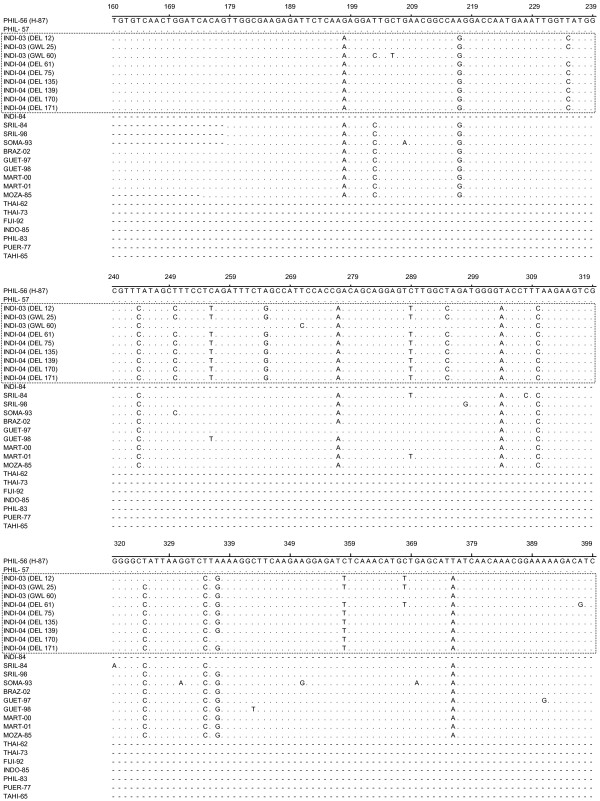
Multiple sequence alignment of C-prM gene junction [nucleotide 160–399 corresponding to the prototype DEN-3 virus (H-87)]. Dot (.) indicates nucleotide similarities with H-87. Dash (-) indicates sequence not available. Each strain is abbreviated with first four letters of country of origin followed by last two digits of the year of isolation.

**Figure 2:**
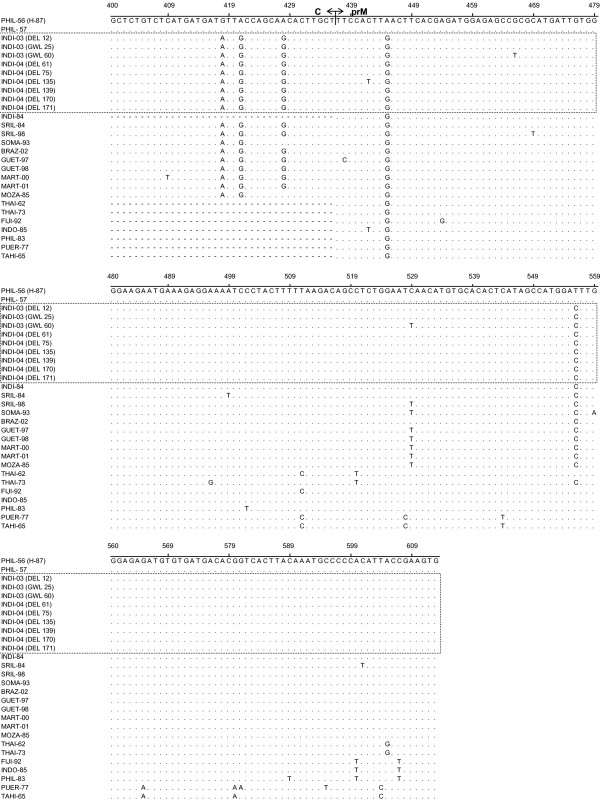
Multiple sequence alignment of C-prM gene junction [nucleotide 400-613 corresponding to the prototype DEN-3 virus (H-87)]. Dot (.) indicates nucleotide similarities with H-87. Dash (-) indicates sequence not available. Each strain is  abbreviated with first four letters of country of origin followed by last two digits of the year of isolation.

**Figure 3:**
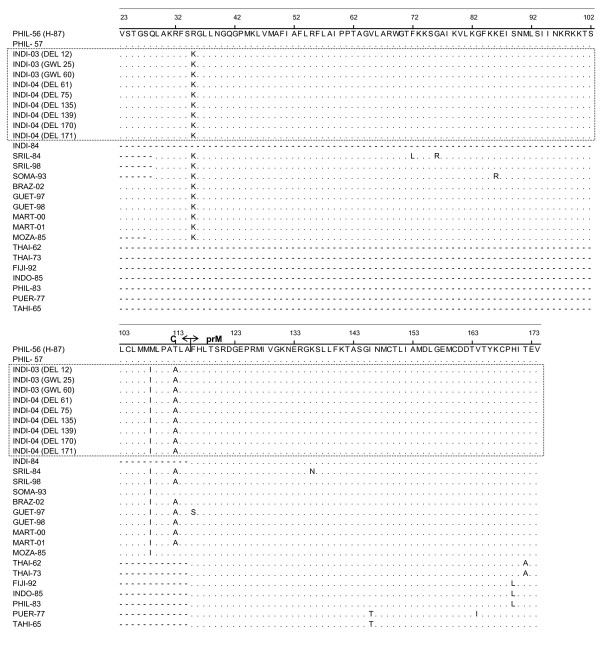
Multiple sequence alignment of deduced amino acid (aa) corresponding to the aa 23 to 173 of the ORF of prototype DEN-3 virus (H-87). Dot (.) indicates amino acid similarities with H-87. Dash (-) indicates sequence not available. Each strain is abbreviated with first four letters of country of origin followed by last two digits of the year of isolation.

The nucleotide sequence of the C-prM gene junction (454 bp; excluding the primer sequence) of the nine representative dengue viruses and one NIV reference DEN-3 virus (isolated in Philippines in 1957) were determined in the present study. Detailed descriptions of these viruses were given in Table 2. These sequences were compared with eighteen other geographically diverse dengue-3 isolates (Table 3). All these sequences were aligned with the homologous regions (nt 160–613) of prototype DEN-3 isolate (H-87, isolated in 1956 in Philippines; designated as PHIL-56 in this manuscript) (Fig. 1 and 2 ). The alignment did not reveal any base insertion or deletion in this region. This region was found to be AT rich and the AT composition of the nine Indian DEN-3 viruses, sequenced in this study varied from 52.42–53.3 % (avg. 52.92 %). On comparison to PHIL-56 (H-87), majority of mutations were found to be silent. Majority of mutations were found to be of transition type. The ratio of transition to transversion was found to be 15:1. The deduced amino acids were also aligned following the nucleotide alignment pattern (Fig 3). Majority of the amino acid changes are found to be conservative type except a very few like M-I (at position 108) and T-A (at position 112). On comparison of C-prM genomic region, it has been found that all the DEN-3 viruses, sequenced in this study, were very closely related (more than 99%), except the GWL-60, which revealed around 97 % nucleotide sequence identity. However, these Indian DEN-3 viruses revealed an average of 95.3% sequence identity with prototype DEN-3 isolate (H-87). When compared with another Indian DEN-3 virus (isolated in 1984), the nucleotide and amino acid sequence homology was found to be 99.84 and 100 % respectively.

**Table 2:** Description of dengue type-3 viruses sequenced in this study

Sl. No	Virus ID. No	Date of collection of sample	Clinical Status	Age (Year)	Sex	Passage History	GenBank Accession No
1	DEL-12	24-10-2003	IU^a^	IU^a^	IU^a^	NIL	AY770513
2	GWL-25	03-11-2003	DF	12	F	NIL	AY770511
3	GWL-60	26-11-2003	DHF	9	M	NIL	AY770512
4	DEL-61	29-09-2004	DHF	22	F	NIL	DQ323037
5	DEL-75	24-09-2004	DF	11	F	NIL	DQ323038
6	DEL-135	16-10-2004	DHF	24	M	NIL	DQ323039
7	DEL-139	16-10-2004	DF	22	F	NIL	DQ323040
8	DEL-170	22-10-2004	DHF	30	F	NIL	DQ323041
9	DEL-171	22-10-2004	DF	22	F	NIL	DQ323042
10	PHIL-57^b^	IU	IU^a^	IU^a^	IU^a^	SM^c ^(P-58)	NS^d^

IU^a^: Information Unknown, ^b ^: NIV Reference Strain

SM^c ^: Suckling mouse, NS^d^: Not submitted

**Table 3:** Description of global dengue-3 viruses used for comparison of genome sequence

Sl. No	Virus ID. No	Year of isolation	Country of origin	Genotype	GenBank Accession No
1	H-87	1956	Philippines	I	M93130
2	A68.AP-2	1983	Philippines	I	L11432
3	85–159	1985	Indonesia	I	L11428
4	29472	1992	Fiji	I	L11422
5	5987	1962	Thailand	II	L11440
6	CH53489	1973	Thailand	II	L11626
7	1416	1984	India	III	L11424
8	BR-74886	2002	Brazil	III	AY679147
9	GUATE97-5	1997	Guatemala	III	AB038473
10	GUATE98-5	1998	Guatemala	III	AB038478
11	H/IMTSSA/1706	2000	Martinique	III	AY099339
12	H/IMTSSA/2012	2001	Martinique	III	AY099340
13	Mozambique85	1985	Mozambique	III	AY665402
14	SOMO79	1993	Somalia	III	AF547240
15	D1440	1984	Sri Lanka	III	AF547229
16	K1	1998	Sri Lanka	III	AF547243
17	65	1965	Tahiti	IV	L11439
18	1340	1977	Puerto Rico	IV	L11434

### Phylogenetic analysis

**Figure 4:**
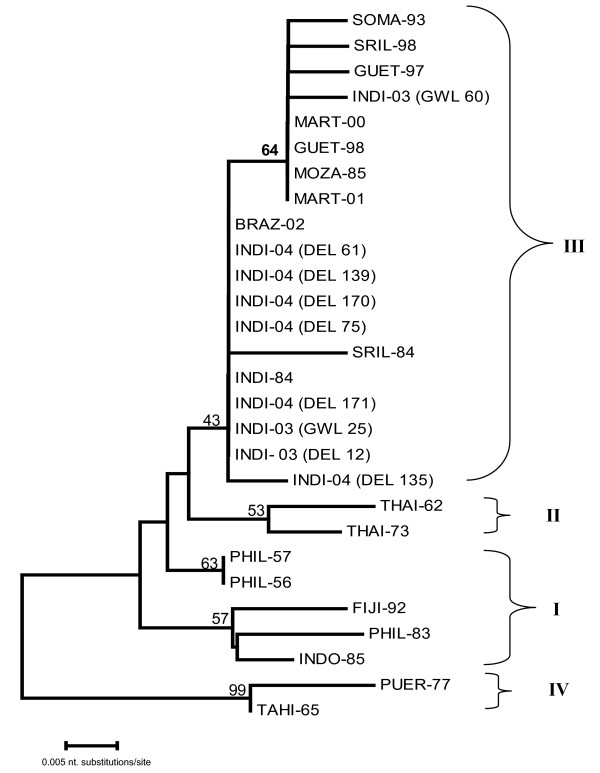
Phylogenetic tree among dengue viruses generated by Neighbour - joining method based on the nucleotide sequence of partial prM gene. Each strain is abbreviated with first four letters of country of origin followed by last two digits of the year of isolation. Bootstrap values are indicated at the major branch points.

**Figure 5:**
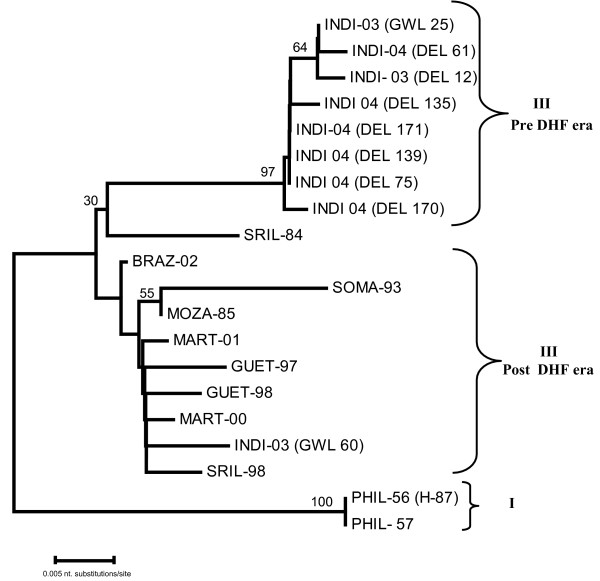
Phylogenetic tree among dengue-3 viruses generated by Neighbour - joining method based on the nucleotide sequence of C-prM gene junction. Each strain is abbreviated with first four letters of country of origin followed by last two digits of the year of isolation. Bootstrap values are indicated at the major branch points. .

Two different dendrograms were drawn based on the pair-wise comparison of nucleotide sequence of partial prM sequence (nt. position 437–613) (Fig 4) and C-prM gene junction (nt. position from 179 to 613, corresponding to PHIL-56) (Fig 5). The dendrogram based on prM clearly revealed four different subtypes of DEN-3 viruses. All the 2003 Indian isolates were grouped into subtype III, along with another Indian DEN-3 virus, isolated in 1984, and large number of isolates recovered from various parts of world, including Asia, Pacific Islands and South American countries. The prototype H-87 (PHIL-56) was found to belong to subtype I, along with two more isolates from Philippines (1957 & 1983) and one each from Indonesia (1990) and Fiji (1992). Two isolates from Thailand (isolated in 1962 and 1973) were found to belong to subtype II, where as two isolates (TAHI-65 and PUER-77) were found to belong to subtype IV.

The dendrogram based on the 435 nucleotide sequence of C-prM gene junction also clearly distinguished the two different genotypes (I and III). All the 2003 and 2004 Indian viruses except GWL-60 form a close branch along with SRIL-84; where as, GWL-60 forms a branch with SRIL-98 and GUET-98 (Fig. 5).

## Discussion

Dengue is now emerging as the most important arboviral infection in most parts of south east Asia including India. In the past, the larger and severe outbreaks in India were mostly caused by dengue virus type-2. However, the investigation of 2003 dengue outbreak in northern India (carried out by us) revealed the involvement of DEN-3 [6] and was also in agreement with another study [7]. Again during the month of September in 2004, dengue reappeared in Delhi and its adjoining areas. Like previous outbreaks, this also struck following the monsoon season, when the climatic factors (temperature and humidity) remained conducive for *Aedes *breeding. The post monsoon dengue outbreak is a regular feature of dengue activity in Indian subcontinent [5-7]. This outbreak was subsided in November upon arrival of winter, when the climatic factors become unfavorable for virus transmission. During this outbreak it has been observed that majority of the patients belonged to age group of > 25 years. However, till date, children and adolescents were recognized as the primary victim of dengue infection [8]. The appearance of dengue primarily among higher age group during this outbreak, suggests the shifting trend towards higher age group. This trend needs to be carefully monitored during ensuing years, as, this can play vital role in planning the control measures.

The routine laboratory diagnosis of dengue virus infection is primarily achieved by the isolation of virus, detection of IgM/IgG antibodies by serodiagnosis and/or molecular detection by the demonstration of viral RNA by RT-PCR [9,10]. In the present study, we screened all the serum samples for the presence of IgM and IgG antibodies by an in house developed Dipstick ELISA assay. This test has been extensively evaluated with field sera collected from different parts of India and can discriminate primary and secondary dengue infection effectively [6,11]. The results of dipstick ELISA assay also supports the dengue viral etiology of the present outbreak. The presence of only IgG antibodies in the majority (51%) of the patients revealed that they were suffering from secondary dengue infection. This is quite expected, as northern India, particularly Delhi is endemic and has witnessed a number of large dengue epidemics in the past decade [4,6,7].

RT-PCR is one of the most important confirmatory test, employed to confirm dengue infection. However, it is only positive when sample is collected during the viraemic phase of the patient (preferably within first five days of onset of symptoms). In the present study, the identification of only 17 samples as dengue positive by RT-PCR, may be due to the fact that most of the patients were presented to the hospitals in the post-viremic phase. Further the lower rate of virus isolation may be attributed to the absence of live virus in the sample. This may be due to failure in maintenance of cold chain resulting in inactivation of virus. All the isolation was confirmed and identified by RT-PCR and nested PCR. Isolation and identification of virus from the clinical sample is considered the gold standard and gives a confirmatory diagnosis without any ambiguity [9,12].

Further, we have carried out the molecular epidemiology and genotyping study of the DEN-3 viruses, implicated as the causative agent of this outbreak. We have studied the sequence of these dengue viruses, directly from patient serum sample, as recently advocated by several researchers [13,14]. Various genomic regions of dengue viruses have been selected for molecular phylogenetic analysis [14-17]. However, we have selected the C-prM gene junction as it also harbours epidemiologically important sequence information. In addition, it provides an economic alternative, since a single set of primer pair could be used for amplification and sequencing of any of the four serotype of dengue virus. Based on C-prM sequence we have earlier reported the circulation of genotype IV of DEN -2 in northern India [14].

On comparison of the sequence, it was found that all the Indian sequences were very closely related. It was found that the outbreak over fairly large areas of two provinces (Delhi and Madhya Pradesh, more than 300 km apart) were caused by the same type of DEN-3 virus. This indicates that the current virus is easily transmitted in human and mosquitoes and can adapt to a newer area efficiently. We have drawn two different dendrograms to study the evolutionary relationship of Indian DEN-3 isolates, due to absence of sequence of capsid gene of any of the DEN-3 viruses belonging to subtype II and IV. One phylogenetic tree was drawn based on the 177 nucleotide sequence of partial prM gene, classified all the 28 DEN-3 viruses, analysed in this study into respective subtypes, as designated in the classic paper of Lanciotti [16]. All the Indian isolates were found to belong to subtype III along with another Indian strain of 1984 and a large number of geographically diverse strains. The dendrogram based on the 435 nucleotide sequence of C-prM gene junction also clearly distinguished the two different subtypes (I and III). All the 2003 and 2004 Indian viruses, except GWL-60 were found to be very closely related and form a close branch along with SRIL-84; where as, GWL-60 form a close branch with SRIL-98 and GUET-98. In an earlier study, based on BLAST search, the DEN-3 viruses from 2003 Delhi outbreak, revealed close genomic homology with GUET-98 [7]. The critical examination of the branching pattern revealed that though all the Indian DEN-3 viruses are closely related to SRIL-84, however they are following a different evolutionary pattern, away from the SRIL-84. It has earlier been reported that SRIL-84 was isolated prior to emergence of DHF in Sri Lanka, where as SRIL-98 was isolated in post DHF era [18].

The dendrogram and sequence analysis clearly revealed that the subtype III of DEN-3 viruses are circulating through out the world, where as other subtypes are localized to a particular small geographical area. This indicates the higher potential of subtype III to spread, adapt and dominate in geographically diverse areas of the world. This subtype has also been implicated in major dengue/DHF epidemic from several parts of Asia, Africa and Americas; and has the potential to cause a trans-national dengue pandemic [18].

Though all the four serotypes of dengue viruses were isolated from different parts of India, DEN-2 was considered as the predominant serotype circulating in northern India [4-7]. DEN-3 associated outbreak was last reported in India in 1994 [19]. However, DEN-3 has been reported as the etiology of the first major DHF outbreak in neighboring Bangladesh in 2001 [20] and also implicated in various outbreaks in Sri Lanka in recent past [18,21]. The identification of the subtype III of dengue-3 from the present outbreak in northern India in 2003 and its continued dominance again in 2004 indicates the resurgence of dengue-3 in a dominant form. The emergence of a newer dengue serotype after an interval always leads to a major outbreak, which is a matter of great concern from public health prospective [22].

## Conclusion

This study confirms that the major dengue outbreak in northern India in 2003 and 2004 was caused by dengue virus type-3 (subtype III). The reemergence of highly fatal subtype III of DEN-3 in a dominant form, replacing the earlier circulating subtype IV of DEN-2 in India is a matter of great concern. Detailed and continuous epidemiological surveillance is warranted to monitor the incursion and spread of dengue viruses, which will help to undertake effective control and management strategies at the earliest.

## Methods

### The outbreak

An outbreak of febrile illness was reported in Delhi, India, during September-October, 2004. A total of 162 blood samples from clinically suspected dengue patients were collected from Delhi during this period. In addition, three viraemic blood samples collected from Delhi and Gwalior during October – December, 2003 [6] were also used in this study for genetic analysis. (Informed consent from all the patients and/or their parents (in minors) were obtained, before collection of clinical samples).

### Serosurveillance

All serum samples were tested for the presence of dengue specific IgM and IgG antibodies using the dengue dipstick ELISA kit developed in our laboratory [11]. Briefly, projections of nitrocellulose (NC) comb (Advanced Microdevices, Ambala, India) were coated with sucrose gradient purified cell culture adapted DEN 1–4 cocktail antigen. For the detection of IgM antibodies, IgG antibodies were first removed from patient sera following adsorption with Protein 'A' derived from *Staphylococcus aureus *Cowan I, where as for detection of IgG antibodies, patient sera were used as such without any pre treatment. Goat anti-human IgM horshradish peroxidase (HRP) and goat anti-human IgG HRP conjugate (Sigma, USA) were used as secondary antibodies for the detection of IgM and IgG antibodies, respectively. The reaction was finally developed by dipping the projections in an insoluble substrate solution (phosphate citrate buffer pH 4.5, containing 3, 3'- diamino benzidine (DAB), 4-chloro-1-napthol and hydrogen peroxide). The results were visually recorded as filled brown dots, indicative of the presence of dengue specific antibodies.

### RT-PCR

The identification of the virus isolates obtained from the clinical samples was carried out by RT-PCR followed by nested PCR by demonstrating the presence of virus specific RNA employing dengue group-specific as well as serotype-specific primers targeting C-prM gene junction following the protocol of Lanciotti *et al*, 1992, with slight modifications [6,23]. Briefly, viral RNA was extracted from 140 μl of serum samples using QIAamp viral RNA mini kit (Qiagen, Germany) in accordance with the manufacturer's instructions and finally RNA was eluted in 50 μl of nuclease free water. The complementary DNA (cDNA) was synthesized in a 10 μl reaction volume with RT mix comprising of 5X-RT buffer, dNTPs, RNasin^® ^ribonuclease inhibitor and Moloney murine leukemia virus reverse transcriptase (MMLV-RT) (Promega, USA) with dengue virus complex specific antisense primer (D2) (Operon, Germany). The RT mix was incubated for 1 h at 37°C, before heating the reaction for 5 min at 99°C to inactivate the MMLV-RT enzyme. The amplification of cDNA was carried out in a total volume of 50 μl with PCR mix containing 10× PCR buffer, 25 m*M *MgCl_2_, NTPs, *Taq*-DNA polymerase (Promega, USA), using dengue virus complex specific sense primer (D1), (Operon, Germany) in a thermal cycler (BioRad, USA). The thermal profile of the PCR reaction was- initial denaturation at 95°C for 2 min., followed by 35 cycles of denaturation at 95°C for 1 min, annealing at 54°C for 1 min, extension at 72°C for 2 min and final extension at 72°C for 10 min. The PCR products were gel purified from 1.2% agarose gel using the QIAquick PCR purification kit (Qiagen, Germany).

### Virus isolation

Isolation of viruses from the acute phase viraemic samples was also attempted in the C6/36 cells, following the standard virus adsorption protocol [12]. Briefly, 500 μl of plasma samples (diluted 1:10 in sterile phosphate buffered saline) was inoculated onto confluent monolayers of C6/36 cells in 25 cm^2 ^tissue culture flasks. The inoculum was incubated for 2 h before being replenished by 10 ml of fresh maintenance medium (Eagles minimum essential medium (EMEM, Sigma) with 10% foetal bovine serum (FBS, Sigma). Suitable healthy cell controls were also kept along side. The cells were then incubated at 32°C and observed microscopically daily for the appearance of cytopathic effects (CPE), if any. The supernantants of infected cell culture were collected on the 6^th ^– 7^th ^post infection day (PID) and analysed for the presence of virus. Invariably, three subsequent serial blind passages were given in each case.

### Sequencing reaction

Double stranded sequencing of the C-prM gene junction was performed on an ABI 310 sequencer (Applied Biosystems, USA), employing Big dye terminator cycle sequencing ready reaction kit. Briefly, 2 μl (approximately 25 ng) of purified PCR product was mixed with 3.2 pmol of respective primer and a reaction mixture containing the four dye-labeled dideoxynucleotide terminators. Cycle sequencing was then performed as follows: 25 cycles at 96°C for 30 sec, 50°C for 1 min and 60°C for 4 min. The reaction mixture was purified by ethanol precipitation and the DNA was vacuum dried. The DNA pellet was resuspended in 10 μl of template suppression reagent (TSR) and preheated before loading on to the DNA sequencer.

### Sequence analysis

The nucleotide sequences were edited and analysed by using the EditSeq and MegAlign modules of the Lasergene-5 software package (DNASTAR Inc, USA). Multiple sequence alignments was done employing CLUSTALW version 1.83 [24]. The phylogenetic tree was constructed by the Neighbour-joining method using MEGA v2.1 programme [25]. The tree topologies were evaluated using 10,000 replicates of the data set.

## Competing interests

The author(s) declare that they have no competing interests.

## Authors' contributions

PKD conceived the study, carried out the sequencing experiments and phylogenetic analysis and drafted the manuscript. MMP carried out the RT-PCR experiments, PS carried out the virus isolation experiments, AA carried out the clinical sample processing, immunoassays and sequence analysis study. CPS and KNT were responsible for collection and storage of clinical samples and meticulous collection of case history. AMJ and KS liaison between MCD and DRDE and coordinated this study. PVLR helped out to design and draft the manuscript and also revised it critically. All authors read and approved the final manuscript.
